# Linking photosynthesis and leaf N allocation under future elevated CO_2_ and climate warming in *Eucalyptus globulus*

**DOI:** 10.1093/jxb/erw484

**Published:** 2017-01-07

**Authors:** Robert E. Sharwood, Kristine Y. Crous, Spencer M. Whitney, David S. Ellsworth, Oula Ghannoum

**Affiliations:** 1 Research School of Biology, Australian National University, Canberra, ACT 2601, Australia; 2 ARC Centre of Excellence for Translational Photosynthesis, Australia; 3 Hawkesbury Institute for the Environment, University of Western Sydney, Locked Bag 1797, Penrith, NSW 2751, Australia.

**Keywords:** Canopy position, elevated CO_2_ and temperature, *Eucalyptus globulus*, photosynthesis, Rubisco kinetics, *V*_cmax_, whole-tree chambers.

## Abstract

Leaf-level photosynthetic processes and their environmental dependencies are critical for estimating CO_2_ uptake from the atmosphere. These estimates use biochemical-based models of photosynthesis that require accurate Rubisco kinetics. We investigated the effects of canopy position, elevated atmospheric CO_2_ [eC; ambient CO_2_ (aC)+240 ppm] and elevated air temperature (eT; ambient temperature (aT)+3 °C) on Rubisco content and activity together with the relationship between leaf N and *V*_cmax_ (maximal Rubisco carboxylation rate) of 7 m tall, soil-grown *Eucalyptus globulus* trees. The kinetics of *E. globulus* and tobacco Rubisco at 25 °C were similar. *In vitro* estimates of *V*_cmax_ derived from measures of *E. globulus* Rubisco content and kinetics were consistent, although slightly lower, than the *in vivo* rates extrapolated from gas exchange. In *E. globulus*, the fraction of N invested in Rubisco was substantially lower than for crop species and varied with treatments. Photosynthetic acclimation of *E. globulus* leaves to eC was underpinned by reduced leaf N and Rubisco contents; the opposite occurred in response to eT coinciding with growth resumption in spring. Our findings highlight the adaptive capacity of this key forest species to allocate leaf N flexibly to Rubisco and other photosynthetic proteins across differing canopy positions in response to future, warmer and elevated [CO_2_] climates.

## Highlight

Photosynthetic acclimation of *Eucalyptus globulus* leaves to elevated CO_2_ was underpinned by reduced leaf N and Rubisco contents; the opposite occurred under warming, coinciding with growth resumption in spring.

## Introduction

Photosynthetic CO_2_ assimilation by the terrestrial biosphere constitutes the largest component of global CO_2_ fluxes. These photosynthetic processes and their responses to the environment are represented in the widely used Farquhar–von Caemmerer–Berry (FvCB) model (Farquhar *et al.*, 1980), which is at the core of most global CO_2_ flux and vegetation productivity models (Zhang *et al.*, 2012; Rezende *et al.*, 2016). The accuracy of the FvCB model is heavily reliant on correct kinetic parameterization of the CO_2_-fixing enzyme Rubisco (ribulose 1,5-bisphosphate carboxylase, EC: 4.1.1.39) as well as knowledge of *J*_max_, the maximum rate of ribulose bisphosphate (RuBP) regeneration (Farquhar *et al.*, 1980). Historically, kinetic surveys of vascular plant Rubisco have generally focused on those from crop and herbaceous species (Kapralov *et al.*, 2011; Hermida-Carrera *et al.*, 2016; Prins *et al.*, 2016) and not for woody plants despite their dominant influence on global net primary production (McGuire *et al.*, 2001; Sitch *et al.*, 2003). Whether the kinetics of crop Rubisco can be used to simulate the rates of photosynthesis accurately in tree species via the FvCB model remains uncertain.

A key component of the FvCB model is the parameter *V*_cmax_, the maximum rate of carboxylation by Rubisco. In particular, *V*_cmax_ is recognized as the most critical parameter for modelling global primary productivity and projecting future global change (Rogers, 2014). This importance stems from estimates of *J*_max_ often being extrapolated from a linear function of *V*_cmax_ (Walker *et al.*, 2014) and that many global carbon models estimate *V*_cmax_ as a fraction of leaf N content (Friend, 2010). Accordingly, the ability to determine *V*_cmax_ using biochemical and leaf photosynthetic measurements for a globally important woody plant genus such as *Eucalyptus* emerges as a key goal to be addressed. Eucalypts are important plants for both native forests and commercial plantations in Australia and worldwide.

According to the FvCB model (Farquhar *et al.*, 1980), *V*_cmax_ is fitted using the initial slope of the rate of CO_2_ assimilation (*A*) versus intercellular [CO_2_] (*C*_i_) response (*A*–*C*_i_) curve, and can be expressed as:

$$V_{\text{c}}=\frac{\text{(}C_{\text{c}}-\Gamma^{\text{*}}\text{)}\times V_{\text{cmax}}}{\text{(}C_{\text{c}}\text{+}K_{\text{c}}{}^{\text{21\%O2}}\text{) }}-R_{\text{d}}$$ (1)

where *V*_c_ is the CO_2_-limited photosynthetic rate, *C*_c_ is chloroplastic [CO_2_], *K*_c_^21%O2^ is Rubisco’s apparent Michaelis–Menten constant for CO_2_ in air, Γ* is the CO_2_ compensation in the absence of mitochondrial respiration (*R*_d_) calculated as 0.5×*O*_c_/*S*_c/o_, with *O*_c_ and *S*_c/o_ representing chloroplastic [O_2_] and Rubisco’s CO_2_/O_2_ specificity, respectively.

In most C_3_ photosynthesis gas exchange studies, *A*–*C*_i_ response curves are fitted with the FvCB model using ‘standard’ catalytic parameters measured for tobacco or spinach Rubisco (Wullschleger, 1993; Badger *et al.*, 2000; Sharkey *et al.*, 2007; Bernacchi *et al.*, 2009). However, significant variation exists in Rubisco catalysis amongst C_3_ species (von Caemmerer and Quick, 2000; Galmes *et al.*, 2005; Whitney *et al.*, 2011*a*;Galmés *et al.*, 2014; Hermida-Carrera *et al.*, 2016; Orr *et al.*, 2016; Prins *et al.*, 2016), including differences in the temperature response of Rubisco between species (Walker *et al.*, 2013; Hermida-Carrera *et al.*, 2016; Prins *et al.*, 2016). Therefore, questions arise about the accuracy of applying these ‘standard’ Rubisco parameters to universally model C_3_ photosynthesis and whether woody plants differ in these respects from crop and herbaceous plants. Consequently, the first objective of the current study was to compare the compatibility of *V*_cmax_ rates derived *in vivo* from the *A*–*C*_i_ curves with *in vitro* estimates of *V*_cmax_ derived from measures of Rubisco content together with assays of the kinetic properties of *Eucalyptus globulus* Rubisco at the standard temperature of 25 °C.

Nitrogen (N) is a major mineral resource limiting plant growth in many parts of the world. About 75% of leaf N is invested in the photosynthetic apparatus, with an average of 20% invested in Rubisco (Evans and Seemann, 1989). Partitioning of photosynthetic N is strongly influenced by the growth environment (Sage *et al.*, 1987; Terashima and Evans, 1988; Evans and Seemann, 1989). It is well documented that elevated atmospheric [CO_2_] reduces leaf N content in many C_3_ species (Drake *et al.*, 1997; Ainsworth and Rogers, 2007, while the effects of warming or CO_2_×warming responses on leaf N content and partitioning are less clear (Onoda *et al.*, 2005*a*, *b*; Hikosaka *et al.*, 2006; Wang *et al.*, 2012). Given that most leaf N is associated with photosynthesis (Evans, 1989; Nakano *et al.*, 1997), changes in leaf N in response to rising [CO_2_] and temperature will impact the photosynthetic biochemistry. To our knowledge, the question of how elevated [CO_2_] and temperature together will influence the underlying photosynthetic biochemistry and N partitioning has not been addressed in large, field-growing tree species. Hence, the second objective of this study was to establish whether *V*_cmax_ constitutes a constant fraction of leaf N under current and future climate conditions.

Only a few studies have investigated the effects of warming on photosynthetic biochemistry and leaf chemistry relative to the large body of work on the effects of elevated [CO_2_] alone. In addition, the interactive effects of climate factors with canopy position is under-represented in the literature (Crous and Ellsworth, 2004). Canopy position is known to influence leaf morphology and chemistry (Ellsworth and Reich, 1993; Kenzo *et al.*, 2006). For example, upper canopy leaves can show the classical sun phenotype whereby a greater proportion of leaf N is allocated to soluble proteins, including Rubisco, and less to thylakoid complexes, including PSII (Boardman, 1977; Givnish, 1988). By addressing the two above-outlined objectives, the current study sought to elucidate the interactive effects of elevated [CO_2_], elevated temperature, and canopy position on determinants of *V*_cmax_ in large, soil-rooted eucalypt trees grown in whole-tree chambers (WTCs) at the Hawkesbury Forest Experiment (HFE) in Richmond, Sydney.

## Materials and methods

### Plant culture and growth conditions

Seedlings of *Eucalyptus globulus* Labill. ssp. were obtained from a commercial tree nursery (Elders Forestry Ltd, Albany, Vic., Australia). Seeds (No. 08-12-106M) were collected at 38°48'S and 143°37'E, ~700 km and five latitudinal degrees pole-ward relative to the experimental site (HFE) of this study (Crous *et al.*, 2013). The HFE site is situated on the alluvial floodplain of the Hawkesbury River (33°36'40''S and 150°44'26.5''E). The soil is a loamy-sand with low organic matter content (0.7%) and low fertility (pH 5.5, N <1 mg kg^–1^, and P ~8 mg kg^–1^). Seedlings were exposed to their respective treatment conditions in the WTCs in August 2010. One tree seedling was transplanted into the ground of each WTC in December 2010 and supplied with an initial fertilization of 50 g of (NH_4_)_2_PO_4_ and 10 mm of water every third day to ensure good establishment. Thereafter, trees were watered every 3–7 d to maintain non-limiting water supply, and were harvested when 10 m tall at 15 months in November 2011. The chamber trees were grown in a continuous block of forest in order to simulate shading by neighbouring trees normally experienced in a canopy.

The WTCs used for this study were designed continuously to track outside (ambient) conditions of temperature, humidity, and [CO_2_] (Barton *et al.*, 2010), and were equipped with improved temperature control (Crous *et al.*, 2013). There were 12 WTCs maintained at four treatments (three WTCs per treatment): ambient [CO_2_] and ambient temperature (aCaT; tracking ambient CO_2_ and ambient temperature); elevated [CO_2_] (tracking ambient CO_2_+240 µl l^–1^) and ambient temperature (eCaT); ambient [CO_2_] and elevated temperature (tracking ambient temperature+3°C) (aCeT); and elevated [CO_2_] and elevated temperature (eCeT). Measurements were made in the early spring of 2011 (August–September 2011) on juvenile, lower and upper canopy leaves of ~7 m tall trees. Only two replicate trees remained in the eCaT treatment as a result of one tree dying following the heatwave of January 2011 (Crous *et al.*, 2013). The other treatments had three healthy trees. During the study period, average growth conditions were: 453 µl l^–1^ and 634 µl l^–1^ for aC and eC treatments, respectively, and 21.4/5.8 °C and 24.3/8.7 °C (day/night) for aT and eT treatments, respectively.

### Leaf gas exchange

A portable open gas exchange system (LI-6400XT, LI-COR, Lincoln, NE, USA) was used to measure the light-saturated photosynthetic rate (*A*_sat_) of upper and lower canopy leaves. Single-point measurements were taken in early spring between 10:00 h and 14:00 h at a leaf temperature of 25 °C, photosynthetic photon flux density of 1500 µmol m^–2^ s^–1^ (light saturating for *E. globulus*, KY Crous, unpublished data), and growth [CO_2_] (400 µl l^–1^ or 640 µl l^–1^). Leaf-to-air vapour pressure deficit (VPD_l_) varied between 0.8 kPa and 1.2 kPa. Each leaf was allowed to stabilize for 15–20 min in the LI-6400XT leaf chamber before measurements were taken. Upper and lower canopy leaves were measured on each of the 11 WTC trees.

Photosynthetic responses to intercellular [CO_2_] (*A*–*C*_i_ curves) were measured on upper canopy leaves only. Measurements were made using 12 CO_2_ steps at a leaf temperature of 25 °C, 1800 µmol photons m^–2^ s^–1^, and VPD_l_ of 0.8–1.1 kPa. The biochemical model of Farquhar *et al.* (1980) was used to estimate *V*_cmax25_ (maximal RuBP carboxylation-limited rate) and *J*_max25_ (maximal RuBP regeneration-limited rate) at 25 °C. The model was parameterized using Rubisco catalytic parameters shown in Table 1 for *E. globulus* and mesophyll conductance (*g*_mes_) for this species obtained from Crous *et al.* (2013).

**Table 1.** Catalytic parameters for *E. globulus* and *Nicotiana tabacum* (tobacco) Rubisco at 25 °C Values (±SD) are the average of the number of biological replicates (*n*) indicated (see the Materials and methods for more detail). The oxygention rate (*k*_cat_^o^) was calculated using the equation *S*_c/o_=(*k*_cat_^c^*/K*_c_)/(*k*_cat_^o^/*K*_o_). Γ^*^, the CO_2_ compensation in the absence of mitochondrial respiration (*R*_d_), was calculated as =0.5×[O_2_]_c_/*S*_c/o_, where [O_2_]_c_ is the O_2_ concentration in the chloroplast. Values used for fitting the *A*–*C*_i_ curves (Table 3; Fig. 2) were converted into the gas phase using the solubility constants for CO_2_ (0.0334 M M^–1^ bar^–1^) and O_2_ (0.00126 M M^–1^ bar^–1^) at 25 ^o^C. CE and OE are the derived carboxylation and oxygenation efficiencies, respectively.

	Eucalyptus globulus	Nicotiana tabacum
*K*_m (CO2)_ at 0% O_2_, *K*_c_^0%O2^ (µM)	9.8 ± 0.3 (293 µbar)	9.4 ± 0.1
*K*_m (CO2)_ at 21% O_2_, *K*_c_^21%O2^ (µM)	21 (629 µbar)	19.5
*k*_cat_^c^ (s^–1^)	3.0 ± 0.2 (*n*=6)	3.1 ± 0.2 (*n*=19)
*K*_i (O2),_ *K*_o_ (µM)	220 ± 18 (175 mbar)	236 ± 9
*S*_c/o_ (M M^–1^) (*n*=2)	80.4 ± 0.9 (2131 bar bar^–1^)	82.3 ± 0.3
Γ^*^ (µM)	1.57 (48.6 µbar)	1.54
*k*_cat_^o^ (s^-1^)	0.94	0.83
CE^21% O2^, k^c^_cat_^c^/*K*_m (CO2)_^21% O2^ (mM^–1^ s^–1^)	143	159
OE, *k*^o^_cat_^o^/*K*_i (O2)_ (mM^–1^ s^–1^)	3.8	4.0

### Content measurements of Rubisco, soluble protein, and chlorophyll

Following the single-point gas exchange measurements, replicate leaf discs (0.98 cm^2^) were rapidly frozen in liquid nitrogen then stored at –80 °C until analysed. High concentrations of secondary metabolites in eucalypt leaves are known to reduce the extraction yield of soluble proteins (Warren *et al.*, 2000). Extraction yield from eucalypt leaves was improved by increasing the polyvinylpolypyrrolidone (PVPP) concentration and adding glycerol to the extraction buffer (see Supplementary Fig. S1A, B at *JXB* online). Each leaf disc was extracted in 1 ml of ice-cold eucalypt protein extraction buffer [50 mM EPPS-NaOH pH 8.0, 5 mM DTT, 15 mM NaHCO_3_, 20 mM MgCl_2_, 2 mM EDTA, 4% (v/v) protease inhibitor cocktail (Sigma), 4% (w/v) PVPP, and 20% (v/v) glycerol] using a 2 ml Potter–Elvehjem glass homogenizer kept on ice. Subsamples were taken from the total extract for chlorophyll determination (90 µl) in 80% acetone (Porra *et al.*, 1989) and SDS–PAGE analysis (75 µl) of total leaf protein. The remaining extract was centrifuged at 16 100 *g* for 1 min and the supernatant used for extractable Rubisco and soluble protein determination. Extractable Rubisco content was quantified by the irreversible binding of [^14^C]carboxyarabinitol bisphosphate (CABP) to the fully carbamylated enzyme (Ruuska *et al.*, 1998). Extractable soluble proteins were measured using the Coomassie Plus kit (Pierce) against BSA. To account for non-extractable soluble proteins (precipitated in the pellet due to high secondary metabolites, Supplementary Fig. S1A), an extraction yield was calculated based on the Coomassie stain intensity of Rubisco large subunit (LSu) detected in total leaf protein (i.e. homogenate before centrifugation) and extractable soluble leaf protein (i.e. supernatant) as described below (Supplementary Fig. S1C). There was a strong relationship between the Coomassie intensity of Rubisco LSu and the amount of Rubisco determined by the [^14^C]CABP assay (Supplementary Fig. S1D), indicating that intensity measurements were adequate for determining differences in Rubisco content and calculating the extraction yield. Total Rubisco and soluble protein concentrations were calculated by dividing extractable Rubisco or soluble proteins by the extraction yield:

$$\text{Total Rubisco sites}=\frac{{\text{Rubisco sites using [}}^{\text{14}}\text{C]CABP assay}}{\text{Extraction yield (from Equation 3)}}$$ (2)

The extraction yield obtained for Rubisco in the current study varied between 0.5 and 0.9.

### Extraction yield and immunoblot of Rubisco and PSII

Subsamples of total and extractable soluble protein fractions were mixed with 0.25 vols of 4× LDS buffer (Invitrogen) containing 100 mM DTT, snap-frozen in liquid nitrogen, and stored at –20 °C until analysed. Protein samples were separated by SDS–PAGE at 200 V using TGX Any kD (Bio-Rad) pre-cast polyacrylamide gels in the Mini-Protean apparatus buffered with Tris-glycine SDS buffer (Bio-Rad). Proteins were visualized by staining with Bio-Safe Coomassie G-250 (Bio-Rad) and imaged using the VersaDoc imaging system (Bio-Rad). The extraction yield of leaf protein was determined from the relative band densitometry of the ~52 kDa Rubisco LSu in 4 µl of both the total and extractable soluble protein fractions according to the equation:

$$\text{Extraction yield}=\frac{\begin{array}{l} \text{Coomassie stain intensity of }\\ \text{LSu in}\;\mathrm{extractable}\;\text{soluble proteins} \end{array}}{\begin{array}{l} \text{Coomassie stain intensity of }\\ \text{LSu in}\;\mathrm{total}\;\text{cellular proteins} \end{array}}$$ (3)

For immunoblot analysis, total extracts for each leaf sample were separated by SDS–PAGE as outlined above and transferred at 4 °C to nitrocellulose membranes (0.45 µm; Bio-Rad) using the Xcell Surelock western transfer module (Invitrogen) buffered with 1× Transfer buffer [20×: 25 mM Bicine, 25 mM Bis-Tris, 1 mM EDTA, 20% (v/v) methanol]. After 1 h transfer at 30 V, the membrane was placed in blocking solution [3% (w/v) skim milk powder in Tris-buffered saline (TBS; 50 mM Tris–HCl pH 8, 150 mM NaCl) for 1 h at room temperature with gentle agitation.

Primary antiserum raised against tobacco Rubisco was diluted 1:4000 in TBS, and antiserum raised against PSII-D1 protein was obtained from AgriSera (PsbA, Cat. AS05-084) and diluted with TBS 1:10 000. Primary antisera were incubated with membranes at room temperature for 1 h with gentle agitation before washing three times with TBS. Secondary goat anti-rabbit antiserum conjugated to horseradish peroxidase (HRP; Cat. NEF 812001EA, Perkin Elmer) was diluted 1:5000 in TBS and incubated with the membranes for 1 h at room temperature followed by three washes with TBS. Immunoreactive peptides were detected using the Immun-Star Western C kit (Cat. 170-5070, Bio-Rad) and imaged using the VersaDoc. Leaf PsbA contents were quantified from band densitometry comparison against 0.25–1 pmol of PsbA global protein standard (Cat. 125-10016, AgriSera) using VersaDoc software Quantity 1.

### Measurements of *in vitro* Rubisco catalytic parameters

Rubisco substrate-saturated turnover rate *k*_cat_^c^ and the Michaelis–Menten constants (*K*_m_) for CO_2_ (*K*_c_) and O_2_ (*K*_i_) were determined by ^14^CO_2_ fixation assays at 25 °C as described (Sharwood *et al.*, 2008; Whitney *et al.*, 2011*b*). Leaf samples were extracted in 1 ml of ice-cold eucalypt extraction buffer (no NaHCO_3_) and the soluble protein activated for 7 min at 25 °C in buffer containing 10 mM NaH^14^CO_3_ and 20 mM MgCl_2_ before adding 20 µl to start 0.5 ml assays in 7 ml septum-capped scintillation vials (Perkin Elmer). The assays contained buffer [50 mM HEPES-NaOH (pH 8.2), 10 mM MgCl_2_, 0.5 mM RuBP] and varying concentrations of NaH^14^CO_3_ (0–74 μM). Assays were equilibrated with 0, 10, 15, 20, 25, or 30% (v/v) O_2_ mixed with N_2_ using Wosthoff gas mixing pumps (Whitney *et al.*, 2011*b*), and terminated after 1 min with 0.2 ml of 20% (v/v) formic acid. For CO_2_/O_2_ specificity (*S*_c/o_) measurements, Rubisco was rapidly purified from ~5 g of young fresh leaves as described in Sharwood *et al.* (2008). *S*_c/o_ was measured using the method of Kane *et al.* (1994). Tobacco was used as a reference species for all Rubisco kinetic assays.

To determine the integrity of Rubisco holoenzyme used for activity assays and to confirm the accuracy of [^14^C]CABP Rubisco content measurements, soluble leaf protein was added to 5× native loading buffer [1 M Tris pH 6.8, 80% glycerol, 1% (w/v) bromophenol blue], separated by non-denaturing PAGE at 60 V for 16 h at 4 °C in 4–12% NuPAGE Tris-glycine gels and the ~520 kDa Rubisco holoenzyme visualized by Coomassie staining (Supplementary Fig. S1B).

### Leaf mass per area, nitrogen, and carbohydrate analyses

Following gas exchange, adjacent leaves were sampled from the upper and lower canopy, their area determined using a leaf area meter (LI-3100A, LI-COR), then oven-dried at 70 °C for 48 h, weighed, and ground to a homogenous powder in a ball mill (MM-400, Retsch, Germany). Leaf mass per area (LMA) was calculated and N content was determined on subsamples using a CN analyser (LECO TruSpec, LECO Corporation, Michigan, USA). The allocation of N into Rubisco, PSII, and soluble proteins was calculated by assuming that proteins contain 16% N by mass, with a mol. wt. of 550 kDa and 417 kDa for Rubisco and PSII, respectively (Evans and Seemann, 1989; Ghannoum *et al.*, 2005).

Another set of matching gas exchange leaves were sampled from the upper and lower canopy at dawn (04:00 h), midday (12:00 h), and dusk (16:00 h), frozen in liquid nitrogen before being stored at –80°C until they were freeze-dried, then ground in a ball mill. Total non-structural carbohydrate (TNC) concentration was calculated as the sum of total starch and soluble sugars, which were measured as described in Ghannoum and Conroy (1998).

### Data analysis

Leaf [N], LMA, TNC, photosynthesis, Rubisco, soluble proteins, and chlorophyll were measured on individual leaf samples per WTC tree and canopy level. For all the parameters, three-way ANOVA was used to test the significance of canopy level, growth [CO_2_], and growth temperature. There were three biological replicates per treatment and canopy level (*n*=3 except for eCaT, where *n*=2 due to tree death). For the quantification of Rubisco, soluble proteins, and PSII proteins, two independent extractions were performed per leaf (two analytical replicates per biological replicate).

## Results

### Leaf gas exchange of *E. globulus* at growth CO_2_

**Fig. 1.**
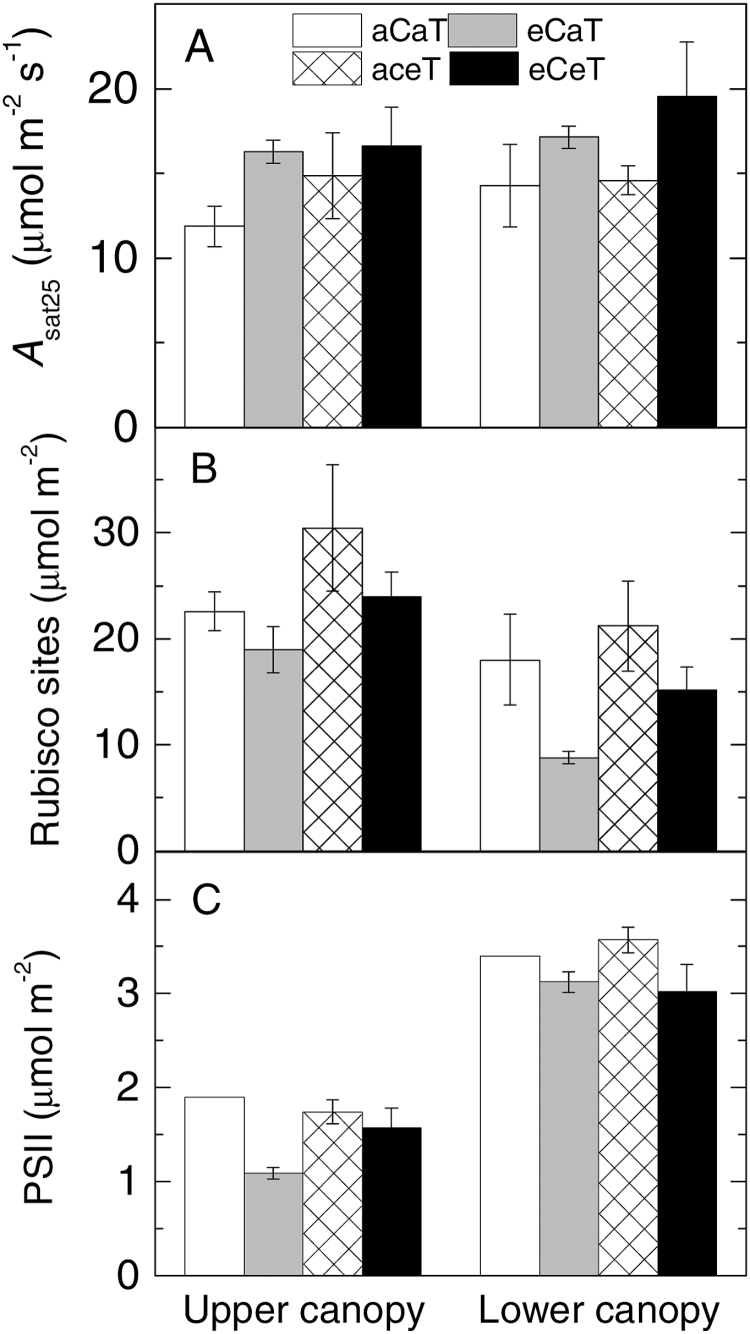
Effect of elevated CO_2_ and temperature on main photosynthetic parameters. Light-saturated rates of photosynthesis, *A*_sat25_ (A), Rubisco content (B), and PSII-D1 protein content (C) in leaves of *E. globulus* trees (upper canopy on the left and lower canopy on the right) grown at ambient (aC, clear and checked columns) or elevated (eC, grey and black columns) atmospheric [CO_2_], and at ambient (aT, clear and grey columns), or elevated (eT, checked and black columns) air temperature. Values represent averages of 2–3 biological replicates ±SE (*n*=3 except for eCaT, where *n*=2). A statistical summary is shown in Table 2.

When measured at a common temperature of 25 °C and growth [CO_2_], leaf photosynthetic rates (*A*_sat25_) of *E. globulus* were stimulated (*P*=0.04) at elevated [CO_2_] by an average of 30% relative to ambient [CO_2_] across both temperatures and canopy positions. In contrast, warming and canopy position had no significant effects on *A*_sat25_ (Fig. 1A; Table 2).

**Table 2.** Summary of three-way ANOVA (canopy×growth [CO_2_]×growth temperature) for leaf parameters measured in *E. globulus* grown at two atmospheric [CO_2_] (ambient and ambient+240 µl l^–1^) and two air temperatures (ambient and ambient+3 ^o^C) Values are means ±SE. Analysis was done with 2–3 biological replicates per treatment and canopy level. There were no significant three-way interactions, and these were not shown. LMA_-TNC_ and Leaf [N]_M-TNC_ were expressed on a structural dry mass basis [i.e. dry mass corrected for TNC (total non-structural carbohydrate) accumulation].

Parameter	Canopy	Treatments				Statistical significance, *P*		
		aCaT (*n*=3)	eCaT (*n*=2)	aCeT (*n*=3)	eCeT (*n*=3)	Canopy Canopy×CO_2_	CO_2_ Canopy×temp	Temp Temp×CO_2_
*A*_sat25_	Upper	11.9 ± 1.2	16.3 ± 0.7	14.8 ± 2.5	16.6 ± 2.2	0.359	**0.042**	0.350
(µmol m^–2^ s^–1^)	Lower	14.3 ± 2.4	17.1 ± 0.7	14.6 ± 0.8	19.5 ± 3.2	0.797	0.916	0.935
Leaf [N]_M_	Upper	15.8 ± 1.9	12.2 ± 0.7	24.7 ± 3.5	18.0 ± 4.0	0.959	**0.025**	**0.0020**
(mg g^–1^)	Lower	17.4 ± 2.5	14.9 ± 0.5	22.2 ± 1.9	15.7 ± 2.0	0.866	0.258	0.376
Leaf [N]_A_	Upper	1.84 ± 0.12	1.85 ± 0.27	2.32 ± 0.22	1.82 ± 0.38	**0.007**	0.283	0.486
(g m^–2^)	Lower	1.47 ± 0.24	1.42 ± 0.10	1.55 ± 0.13	1.35 ± 0.18	0.725	0.520	0.331
PNUE	Upper	92 ± 15	126 ± 23	89 ± 11	133 ± 15	**0.000**	**0.000**	0.444
[µmol (mol N)^–1^ s^-1^]	Lower	136 ± 4	171 ± 19	133 ± 12	201 ± 8	0.531	0.544	0.280
Rubisco sites	Upper	22.5 ± 1.5	18.9 ± 2.2	30.5 ± 5.9	23.9 ± 2.3	0.145	**0.026**	**0.046**
(µmol m^–2^)	Lower	18.0 ± 4.3	8.8 ± 0.6	21.2 ± 4.3	15.1 ± 2.2	0.427	0.345	0.904
Soluble proteins	Upper	5.4 ± 0.8	5.7 ± 1.3	6.8 ± 0.3	6.7 ± 1.9	0.059	0.330	0.323
(g m^–2^)	Lower	5.2 ± 0.7	3.2 ± 1.0	5.1 ± 0.6	4.5 ± 0.8	0.466	0.851	0.903
Chlorophyll	Upper	0.75 ± 0.17	0.57 ± 0.05	0.88 ± 0.07	0.85 ± 0.17	0.222	0.400	0.157
(mmol m^–2^)	Lower	0.62 ± 0.15	0.63 ± 0.04	0.72 ± 0.03	0.63 ± 0.11	0.723	0.395	0.874
LMA	Upper	118 ± 7	152 ± 13	97 ± 11	102 ± 2	**0.000**	**0.002**	**0.000**
(g m^–2^)	Lower	84 ± 2	95 ± 4	70 ± 2	86 ± 3	0.490	**0.015**	0.210
Average daily TNC	Upper	30 ± 1	39 ± 2	20 ± 3	23 ± 1	**0.000**	**0.003**	**0.000**
(g m^–2^)	Lower	17 ± 1	23 ± 3	15 ± 1	16 ± 1	0.296	**0.004**	**0.049**
LMA_-TNC_	Upper	88 ± 5	113 ± 11	77 ± 7	80 ± 1	**0.000**	**0.003**	**0.001**
(g DM-TNC m^–2^)	Lower	66 ± 2	72 ± 1	54 ± 3	70 ± 2	0.615	**0.037**	0.381
Leaf [N]_M-TNC_	Upper	21 ± 3	16 ± 1	31 ± 4	23 ± 5	0.837	**0.023**	**0.031**
[mg g (DM-TNC)^–1^]	Lower	22 ± 3	20 ± 1	28 ± 2	19 ± 2	0.890	0.297	0.317

### Contents of leaf Rubisco, PSII, soluble proteins, and chlorophyll

Leaf Rubisco contents were assayed using two independent techniques: (i) quantification of total Rubisco catalytic sites using tight stoichiometric [^14^C]CABP binding (Table 2) and (ii) confirmatory relative Rubisco content measured by immunoblot analysis of SDS–PAGE-separated Rubisco LSu (Supplementary Fig. S1). The two methods reconciled closely with each other (Supplementary Fig. S1D). Upper canopy leaves tended (*P*=0.14) to have more Rubisco content relative to the lower canopy (Fig. 1B). Elevated [CO_2_] (eC) reduced Rubisco content (*P*=0.03) by 16% and 51% in upper and lower leaves, respectively, while elevated temperature (eT) enhanced Rubisco content (*P*=0.046) by 36% and 18% in upper and lower leaves, respectively (Fig. 1B; Table 2). The effects of eC and eT were additive such that leaf Rubisco content was similar between the eCeT and aCaT treatments (Fig. 1B; Table 2). The leaf soluble protein content tended (*P*=0.059) to be lower in the lower relative to the upper canopy leaves; and was reduced by ~40% in eC in the lower canopy while it was increased by ~25% in eT in the upper canopy (Table 2).

The content of PSII, a key thylakoid protein involved in light harvesting and electron transport, was determined by measuring the abundance of the PsbA (D1) protein by immunoblot analysis using the Agrisera global D1 protein antibody designed to react equally with D1 protein from all plant species. Upper canopy leaves had about half of the PSII content compared with the lower canopy (Fig. 1C). The eC treatment reduced the D1 protein content in the upper canopy leaves but was not significantly affected by eT (Fig. 1C). Despite these changes in PSII, leaf chlorophyll content in *E. globulus* was not significantly affected by either canopy position, growth [CO_2_], or temperature (Table 2).

### Catalytic comparison of *E. globulus* and tobacco Rubisco

The catalytic properties of *E. globulus* Rubisco measured at 25 °C are similar to those of tobacco Rubisco (Table 1). With regard to the parameters used in Equation 1, both Rubisco isoforms show comparable substrate-saturated carboxylation rates (*k*_cat_^c^) while the CO_2_/O_2_ specificity (*S*_c/o_) and carboxylation efficiency [*k*_cat_^c^*/K*_m (CO2)_^21% O2^] of *E. globulus* Rubisco at 21% O_2_ were slightly lower than those of tobacco Rubisco (Table 1).

### Analysis of the *A–C*_i_ curves at 25 ^o^C

**Fig. 2.**
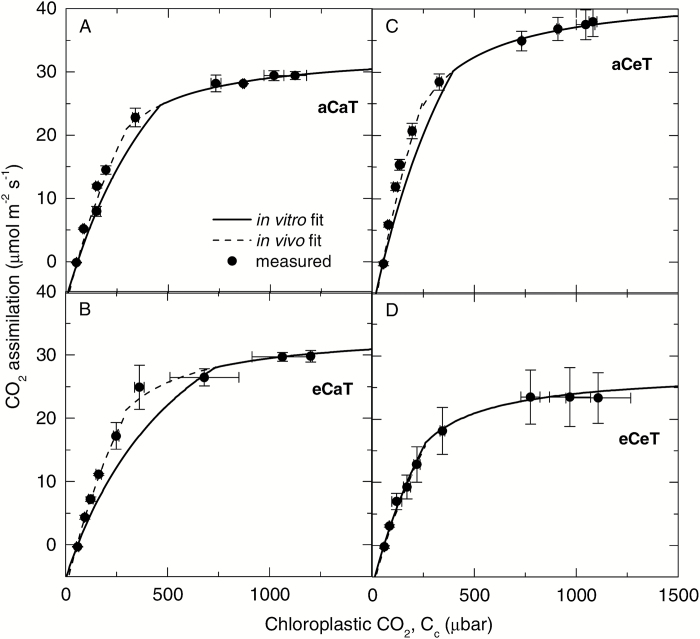
Effects of elevated CO_2_ and temperature on photosynthetic CO_2_ response curves. The response of photosynthetic rates to chloroplastic [CO_2_], *C*_c_ in the upper canopy leaves of *E. globulus* trees grown at ambient (aC) or elevated (eC) atmospheric [CO_2_], and at ambient (aT) or elevated (eT) air temperature. Data points (filled circles) are the average (±SE) *A*–*C*_i_ curve measured at 25 ^°^C for 2–3 biological replicates (*n*=3 except for eCaT, where *n*=2). Lines represent theoretical *A*–*C*_i_ curves modelled using the *in vitro* (solid lines) or *in vivo* (dashed lines) estimates of *V*_cmax_ as described in Table 1.

The CO_2_ response curves of photosynthesis were measured for upper canopy leaves (Fig. 2). Analysis of the *A*–*C*_i_ response curves using catalytic parameters for *E. globulus* Rubisco (Table 1) and *g*_mes_ reported in Crous *et al.* (2013) revealed that elevated [CO_2_] had no significant effect on the *in vivo* gas exchange estimates of either *V*_cmax25_ or *J*_max25_. In contrast, elevated temperature (aCeT) enhanced the *in vivo V*_cmax25_ and *J*_max25_ by 26% relative to the aCaT treatment (Fig. 2; Table 3). The ratio *J*/*V* (1.5–1.7) was not significantly affected by any treatment (Table 3).

**Table 3.** Summary of *in vitro* and *in vivo* estimates of *V*_cmax_ and *J*_max_ for upper canopy leaves of *E. globulus* *In vitro V*_cmax_ was calculated as [Rubisco sites]×*k*_cat_^c^. The biochemical model of Farquhar *et al.* (1980) was used to estimate *in vivo V*_cmax_ (maximal RuBP carboxylation-limited rate) and *J*_max_ (maximal RuBP regeneration-limited rate) from the *A*–*C*_i_ curves measured at 25^o^C for upper canopy leaves. The model was parameterized using Rubisco catalytic parameters shown in Table 1 for *E. globulus* and mesophyll conductance (*g*_mes_) obtained from Crous *et al.* (2013). The equations used were: $V_{\text{c}}=\frac{\text{(}C_{\text{c}}-\Gamma^{\text{*}}\text{)}\times V_{\text{cmax}}}{\text{(}C_{\text{c}}\text{+}K_{\text{c}}{}^{\text{21\%O2}}\text{) }}-R_{\text{d}}$; $V_{\text{j}}=\frac{{\text{ (C}}_{\text{c}}-\Gamma^{\text{*}}\text{)}\times J_{\text{max}}}{\text{(4}C_{\text{c}}-\text{8}\Gamma^{\text{*}}\text{)}}-R_{\text{d}}$ and ${\text{C}}_{\text{c}}={\text{C}}_{\text{i}}-\frac{A_{\text{sat}}}{g_{\text{mes}}}$ where C_c_ is the chloroplastic [CO_2_]. Other parameters are defined in Table 1.

Parameter	aCaT	eCaT	aCeT	eCeT
*In vitro V*_cmax_ (μmol m^–2^ s^–1^)	67 ± 5	57 ± 6	91 ± 18	72 ± 7
*In vivo V*_cmax_ (μmol m^–2^ s^–1^)	81 ± 5	81 ± 15	115 ± 17	72 ± 21
*In vivo J*_max_ (μmol m^–2^ s^–1^)	138 ± 5	140 ± 16	175 ± 17	115 ± 23
*In vivo J/V*	1.7	1.7	1.5	1.6
*V*_cmax_ (*in vivo/in vitro* ratio)	0.8	0.7	0.8	1.0

*In vitro* and *in vivo* estimates of *V*_cmax_ were equal for the eCeT treatment, such that measured leaf Rubisco contents could account for the carboxylase-limited assimilation rates of the *A*–*C*_i_ curve (Fig. 2D; Table 3). For the other treatments, 20–30% more Rubisco sites were required to account fully for the CO_2_ assimilation rates measured *in vivo* under limiting *C*_i_ (Fig. 2A–C; Table 3). This demonstrates the difficulty of achieving high extraction yields for leaf soluble proteins in recalcitrant species, such as eucalypts.

### Leaf nitrogen and carbohydrates

LMA and TNC changed in parallel with the various treatments (Table 2). LMA and TNC were generally greater in upper than in lower canopy leaves (*P*<0.001). In both canopies, LMA and TNC increased at elevated [CO_2_] (*P*=0.002) and decreased at elevated temperature (*P*<0.001) such that values were similar for the aCaT and eCeT treatments (Table 2). Changes in LMA mirrored those observed for TNC-corrected LMA (LMA_-TNC_), indicating that elevated [CO_2_] and temperature affected both structural and non-structural carbohydrates (Table 2).

Expressed on a dry mass basis, leaf nitrogen content ([N]_M_) was similar in both canopy positions (*P*=0.96). Relative to the aCaT treatment, leaf [N]_M_ decreased by 23% at elevated [CO_2_] (*P*=0.03) and increased by 56% at elevated temperature (*P*=0.002); leaves grown at aCaT and eCeT had similar leaf [N]_M_ (Table 2). Similar trends were observed for [N]_M_ when dry mass was corrected for TNC, [N]_M-TNC_ (Table 2). Expressed on an area basis, leaf nitrogen concentration ([N]_A_) was 25% greater in upper than in lower canopy leaves (*P*=0.008). Elevated [CO_2_] and elevated temperature had no significant effect on leaf [N]_A_ either separately or jointly (Table 2).

### Leaf nitrogen relations and its allocation

**Fig. 3.**
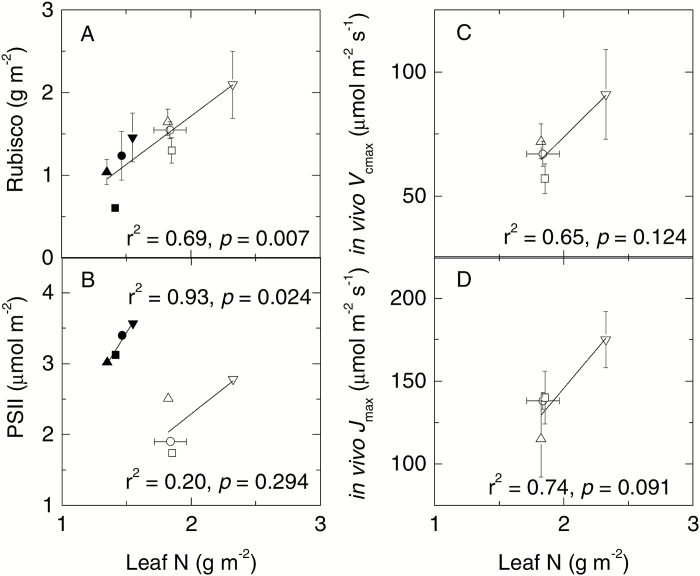
Effects of elevated CO_2_ and temperature on the relationship between leaf photosynthesis and N. Relationships between leaf Rubisco (A) and PSII (B) contents and *in vivo V*_cmax_ (C) and *J*_max_ (D) with leaf N of *E. globulus* trees grown in whole-tree chambers. Values represent the means ±SE of 2–3 biological replicates for upper (open symbols) and lower (filled symbols) canopy leaves grown at aCaT (circles), eCaT (squares), aCeT (inverted triangles), and eCeT (upright triangles). The solid lines are linear fits of the data points.

Leaf contents of Rubisco (Fig. 3A) and soluble proteins (*r*^2^=0.58, *P*=0.035) scaled strongly with leaf [N]_A_ across the treatment combinations. In contrast, leaf PSII content and [N]_A_ scaled better in lower relative to upper canopy leaves (Fig. 3B). *In vivo V*_cmax25_ and *J*_max25_ correlated with leaf [N]_A_ in upper canopy leaves across the [CO_2_] and temperature treatments in *E. globulus* (Fig. 3C, D).

**Fig. 4.**
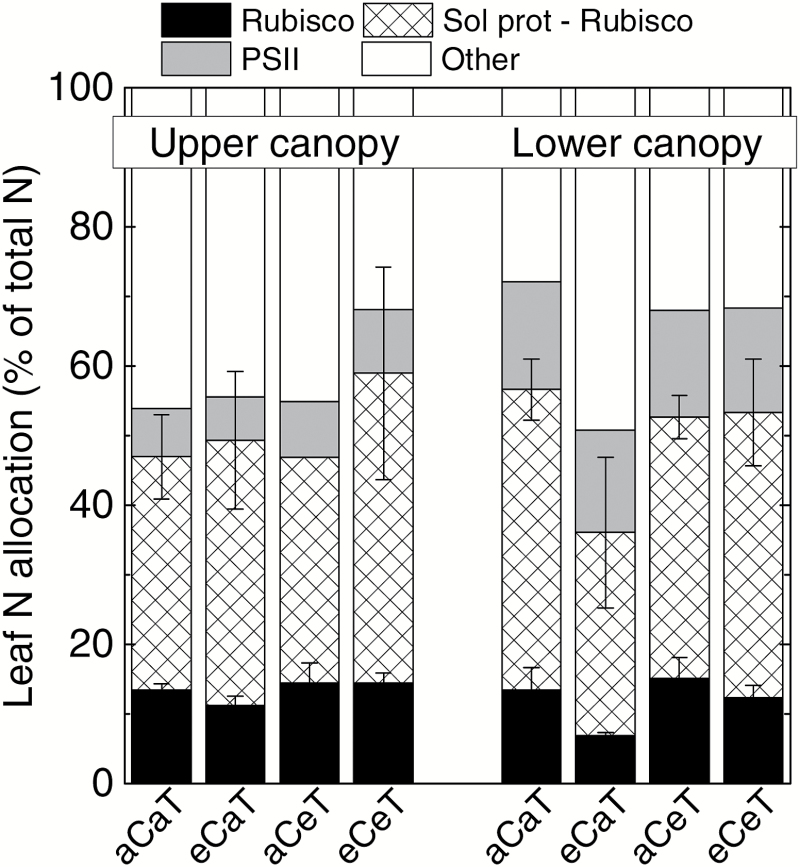
Leaf nitrogen allocation of *E. globulus* in trees grown at ambient (aC) or elevated (eC) atmospheric [CO_2_], and at ambient (aT) or elevated (eT) air temperature. Values represent averages of 2–3 biological replicates ±SE (*n*=3 except for eCaT, where *n*=2). The N percentages were calculated using data in Table 2 and Fig. 1. Other details are as described in the Materials and Methods.

Rubisco constituted 19–31% of the leaf soluble proteins and 7–15% of leaf N *in E. globulus* (Table 2). Relative to the lower canopy, upper canopy leaves invested 2-fold more N in Rubisco, 1.5-fold more N in soluble proteins, and 2-fold less N in PSII (Fig. 4). Either separately or together, elevated [CO_2_] and temperature had no significant effect on the proportion of leaf N invested in soluble proteins, Rubisco, or PSII (Fig. 4).

Upper canopy leaves had 30% lower photosynthetic nitrogen use efficiency, PNUE (*P*=0.0001) compared with the lower canopy (Table 2). Elevated [CO_2_] increased PNUE (*P*=0.0003) by ~30% in non-warmed trees and ~50% in warmed trees, while warming had no significant effect on PNUE (Table 2).

## Discussion

### Estimating *V*_cmax_ using Rubisco parameters

This study presents the first comprehensive attempt at estimating *in vivo* and *in vitro V*_cmax_ for a widely planted tree species (*E. globulus*) grown under varying atmospheric [CO_2_] and temperature using combined measurements of its leaf Rubisco content and inherent Rubisco kinetic properties together with leaf gas exchange. For trees, numerous studies have considered the response of leaf gas exchange to single or multiple climate change variables (Tjoelker *et al.*, 1998; Teskey and Will, 1999; Ellsworth *et al.*, 2004; Crous *et al.*, 2008, 2013; Ghannoum *et al.*, 2010*b*; Quentin *et al.*, 2015). Studies examining the response of photosynthetic proteins or enzyme activity to climate change have largely focused on crop and other non-woody species, and mostly in response to elevated [CO_2_] without warming (Nakano *et al.*, 1997; Rogers *et al.*, 1998; Theobald *et al.*, 1998; Adam *et al.*, 2000). Only a few tree studies have measured changes in leaf Rubisco content (Tissue *et al.*, 1993; Rogers and Ellsworth, 2002; Kosvancova *et al.*, 2009; Urban *et al.*, 2012) with accurate quantification in the leaves of trees such as eucalypts or pines particularly challenging due to high levels of protein-damaging, secondary metabolites (Rogers *et al.*, 2001). Inclusion of glycerol, high amounts PVPP, and a plant protease inhibitor were found to be effective in establishing a method to meet our first objective, the extraction and quantification of active Rubisco from *E. globulus* leaves to derive *in vitro* estimates of *V*_cmax_ at 25 ^o^C. The derived values were consistent, although slightly lower, than the *in vivo* measures of *V*_cmax_ extrapolated from gas exchange (Table 3). In agreement with our finding, similar differences between the *in vitro* and *in vivo* estimates of *V*_cmax_ were reported for loblolly pines (Rogers *et al.*, 2001).

A critical aspect of photosynthesis modelling is the ability to link *V*_cmax_ to easily measured leaf traits such as leaf N content, as well as the relationship between *V*_cmax_ and *J*_max_ (Walker *et al.*, 2014). Data presented in this study demonstrated that Rubisco constituted 7–15% of leaf N across the various treatments. As shown in Table 3, these values are 20–30% lower based on *in vivo V*_cmax_ estimates. Hence, the true Rubisco fraction is expected to be 9–18% of leaf N, which is somewhat lower than the average of 20% generally observed for C_3_ species (Evans, 1989). Nevertheless, *V*_cmax_ values observed for *E. globulus* (57–115 µmol m^–2^ s^–1^) were generally higher than values (<70 µmol m^–2^ s^–1^) recorded for broad-leaf evergreen tree species (Rogers, 2014), suggesting differences in the Rubisco content and/or catalytic properties between these tree species. This is an area worthy of further investigation. Importantly, there was a strong linear relationship between leaf N and Rubisco content (Fig. 3A) across canopy positions. *V*_cmax_ was also well correlated with leaf N in the upper canopy (Fig. 3C), while the *J*_max_/*V*_cmax_ ratio was constant across the various treatments (Table 3). Taken together, these results indicate that in *E. globulus* Rubisco largely remained a constant fraction of leaf N across the elevated CO_2_, warming, and canopy position treatments, and that *V*_cmax_ in this species can be predicted from leaf N and the Rubisco fraction, while *J*_max_ can also be estimated from *V*_cmax_. These are important relationships for whole-tree and canopy scale modelling of CO_2_ fixation (Rogers, 2014; Walker *et al.*, 2014).

### Acclimation in response to elevated [CO_2_] and temperature

In the soil-grown *E. globulus* trees, elevated [CO_2_] increased LMA and reduced leaf [N] expressed on both a total dry mass and structural dry mass basis by the same proportion. In contrast, elevated temperature had the opposite effects on LMA and leaf [N], respectively. In response to eC and eT, leaf N expressed on an area basis was unchanged. These findings correlate with those observed for many other C_3_ species exposed to elevated [CO_2_] in controlled environments or in the field (Tjoelker *et al.*, 1998; Ellsworth *et al.*, 2004; Crous *et al.*, 2008; Ghannoum *et al.*, 2010*a*), and refute carbohydrate dilution as an explanation for reduced leaf [N]_M_ at eC (Taub and Wang, 2008). The effects of eT on leaf [N] in tree species are less well documented and more variable, with reports of an increase for Scots pine (Kellomaki and Wang, 1997) compared with small decreases for sugar maple (Gunderson *et al.*, 2010) and eucalypts (Ghannoum *et al.*, 2010*a*; Sherwin *et al.*, 2013). Similar trends for LMA and leaf TNC have been reported for various tree species in response to warming (Tjoelker *et al.*, 1998; Gunderson *et al.*, 2010). Importantly, in *E. globulus*, changes in leaf N and Rubisco content were linearly related across the various treatments (Fig. 1A) with the leaf D1 content (an indicator of the amount of the thylakoid PSII complex) also shown to scale with leaf N within each canopy position (Fig. 1B). Consequently, changes in Rubisco and PSII contents in response to eC and eT were underpinned by generic shifts in leaf N.

Our findings indicate that the leaf CO_2_ assimilation rates in *E. globulus* grown under both aC and eC remained predominantly RuBP carboxylation limiting (Bowes, 1991; Sage, 1994; Moore *et al.*, 1999; Rogers and Humphries, 2000; Rogers and Ellsworth, 2002). Under eC, leaf Rubisco content was reduced without compromising *V*_cmax25_ (derived *in vivo* from gas exchange) or the short-term stimulation of photosynthetic rates in response to increased [CO_2_] determined at a common temperature (*A*_sat25_). The maintenance of *V*_cmax25_ with less leaf Rubisco content may be due to increased Rubisco activation (i.e. the proportion of active/total Rubisco sites) at eC as noted in other studies using C_3_ species (Salvucci *et al.*, 1986; Sage *et al.*, 1989; Tissue *et al.*, 1993). This finding is in line with the strong biomass stimulations observed for the *E. globulus* trees grown at eC (Quentin *et al.*, 2015). In contrast, eT led to increased leaf [N] of upper canopy *E. globulus* leaves as well as a large increase in *V*_cmax25_ (+40%) and *J*_max25_ (+30%), while *A*_sat25_ did not significantly vary between the temperature treatments. Hence, thermal acclimation expressed as up-regulation of photosynthetic proteins served to sustain photosynthetic rates of *E. globulus* at elevated temperature. This finding concurs with the lack of biomass stimulation observed in this species when grown under eT (Quentin *et al.*, 2015). Moreover, in early spring when the current study was undertaken, leaf photosynthesis was operating near its thermal optimum, ~21 °C (Crous *et al.*, 2013), which is much higher compared with its winter thermal optimum in its native range, ~16.5 °C (Battaglia *et al.*, 1996). Consequently, short-term warming of +3 °C (in the absence of acclimation) may have reduced photosynthetic rates at ambient [CO_2_] by shifting the operating range beyond the thermal optimum. This type of acclimation which elicits a generic up-regulation of leaf N and photosynthetic proteins is described as ‘quantitative’ by Way and Sage (2008) and is contingent on the availability of N resources for increased investment in the photosynthetic apparatus.

### The interactive effects of elevated [CO_2_] and temperature

The effects of eC and eT on photosynthetic components were additive and offsetting such that most measured parameters were similar between the aCaT and eCeT treatments, with the exception of *A*_sat_. Up-regulation of *V*_cmax_ and *J*_max_ in response to eT served to alleviate the negative effects of short-term increases in temperature on photosynthesis when operating close to the thermal optimum. Elevated [CO_2_] shifted the temperature response of photosynthesis upwards and increased its thermal optimum due to increased RuBP carboxylation and decreased RuBP oxygenation (Long, 1991; also reported in Crous *et al.*, 2013). In so doing, eC eliminated the need for the resource-expensive ‘quantitative’ acclimation response under eCeT; while allowing photosynthesis to respond positively to warming. Accordingly, the extent to which the effects of eC and eT on photosynthetic capacity cancel each other out depends on whether warming occurs towards or away from the photosynthetic thermal optimum, in addition to the plasticity of the plant to adjust their thermal optimum seasonally or in response to changes in growth conditions.

### The influence of canopy position

*Eucalyptus globulus* trees examined in the current study were 1.5 years old, ~7 m tall, and with extensive amounts of juvenile foliage. Canopy position influenced leaf morphology and photosynthetic N allocation rather than leaf [N]_M_ or photosynthetic rates. Relative to the lower canopy, the upper canopy in *E. globulus* allocated a greater proportion of their leaf N to soluble proteins, including Rubisco, and less to PSII (Table 2; Figs 1, 4). These differences are in line with the classical sun versus shade phenotypes observed across plant species (Boardman, 1977; Givnish, 1988). Similar results have been observed for other tree species whereby the positional effect is expressed at the level of photosynthetic N allocation to achieve similar photosynthetic rates (Ellsworth and Reich, 1993; Crous and Ellsworth, 2004; Kenzo *et al.*, 2006; Turnbull *et al.*, 2007; Ellsworth *et al.*, 2012; Mao *et al.*, 2012; Weerasinghe *et al.*, 2014). Hence, clear positional effects on leaf traits were observed in *E. globulus* despite the fact that light distribution may be more diffuse within the WTC relative to the open air field (Medhurst *et al.*, 2006; Barton *et al.*, 2010).

One of the objectives of our study was to establish whether the photosynthetic response to eC and eT in *E. globulus* depended on canopy position. It has been hypothesized that within-canopy differences in the light environment may influence the response to eC (Kubiske *et al.*, 2002) as a result of light-driven differences in photosynthetic N allocation (Evans, 1993; Hikosaka and Terashima, 1995). Although lower canopy leaves showed weaker responses to eC and eT relative to upper canopy leaves, there were no significant canopy×treatment interactions for any of the measured parameters (except for a significant canopy×temperature effect on LMA), probably due to the open crown structure in *Eucalyptus* species. In other experiments where there were large positional trends in leaf [N], the response to eC differed with canopy position (Herrick and Thomas, 1999; Crous and Ellsworth, 2004).

### Conclusions

Rubisco catalytic parameters for *E. globulus* measured at 25 °C were similar to the widely used tobacco kinetics in C_3_ photosynthesis, and may provide a model for evergreen plantation trees, although caution is needed in the general applicability of these parameters across different taxa and temperatures (Sharwood *et al*., 2016). Characterization of Rubisco protein content and catalytic parameters enabled *in vitro* estimates of *V*_cmax_ that were consistent, although slightly lower, than *in vivo* rates extrapolated from gas exchange. In *E. globulus*, *V*_cmax_ can be predicted from leaf N and the Rubisco contents, while *J*_max_ can also be estimated from *V*_cmax_.

In response to eC, the leaves of *E. globulus* trees underwent a photosynthetic acclimation underpinned by down-regulation of leaf N and Rubisco contents that improved PNUE. In contrast, there was a generic up-regulation of photosynthetic proteins in eT via increased leaf [N]; this response could be key to the resumption of growth in spring, albeit at an added N cost. The ability of *E. globulus* leaves to allocate leaf N flexibly in response to environmental cues led to opposite and offsetting effects of eC and eT on photosynthetic capacity. Consequently, the biochemical balance of *E. globulus* leaves in the warmer elevated [CO_2_] treatment was not markedly different from that in the current climate. In contrast to the CO_2_ and temperature treatments, canopy position affected the allocation of leaf N to Rubisco and PSII proteins.

## Supplementary data

Supplementary data are available at *JXB* online.

Fig. S1. Determination of Rubisco integrity and extraction yield in *E. globulus* using Coomassie blue staining.

Fig. S2. Relative content per leaf area of the thylakoid complex PSII.

## Supplementary Material

supplementary_figures_S1_S2
