# Supplementary material for: Linking photosynthesis and leaf N allocation under future elevated CO2 and climate warming in Eucalyptus globulus
Source: J Exp Bot. 2017 Jan 7;68(5):1157–67. doi: 10.1093/jxb/erw484 (PMC5444472; doi:10.1093/jxb/erw484)

SUPPLEMENTARY DATA

Linking photosynthesis and leaf N allocation in *Eucalyptus globulus* under future elevated CO_2 and climate warming

Robert E. Sharwood[1,2], Kristine Y. Crous[3], Spencer M. Whitney[1,2], David S. Ellsworth[3], Oula Ghannoum[2,3]*

Supplementary Figure S1: Determination of Rubisco integrity and extraction yield in *E. globulus* using Coomassie blue staining. (A) SDS-page followed by Coomassie staining of proteins extracted from *E. globulus* and tobacco leaves. Subsamples from the total crude (Cr) extracts were separated alongside subsamples taken after centrifugation from the soluble (Sol) and pellet (Pel) fractions. Bands containing Rubisco large (LSu) and small (SSu) subunits are indicated. M, Precision plus protein marker (Bio-Rad) and the MW of the standards from top of gel are 250, 150, 100, 75, 50, 37, 25, 20 , 15, 10 kDa.

(B) An example of a native, non-denaturing PAGE of soluble proteins extracted from tobacco and *E. globulus* leaves. This analysis was undertaken on extracts used to determine total Rubisco sites using the [^{14}C]CABP assay to ensure the integrity of the holoenzyme.

(C) Coomassie staining of proteins extracted from total (crude) and soluble extracts from four *E. globulus* leaf samples.

(D) An example of a standard curve showing a linear relationship between micrograms of Rubisco determined by the [^{14}C]CABP assay and Coomassie band intensity of LSu for *E. globulus*.

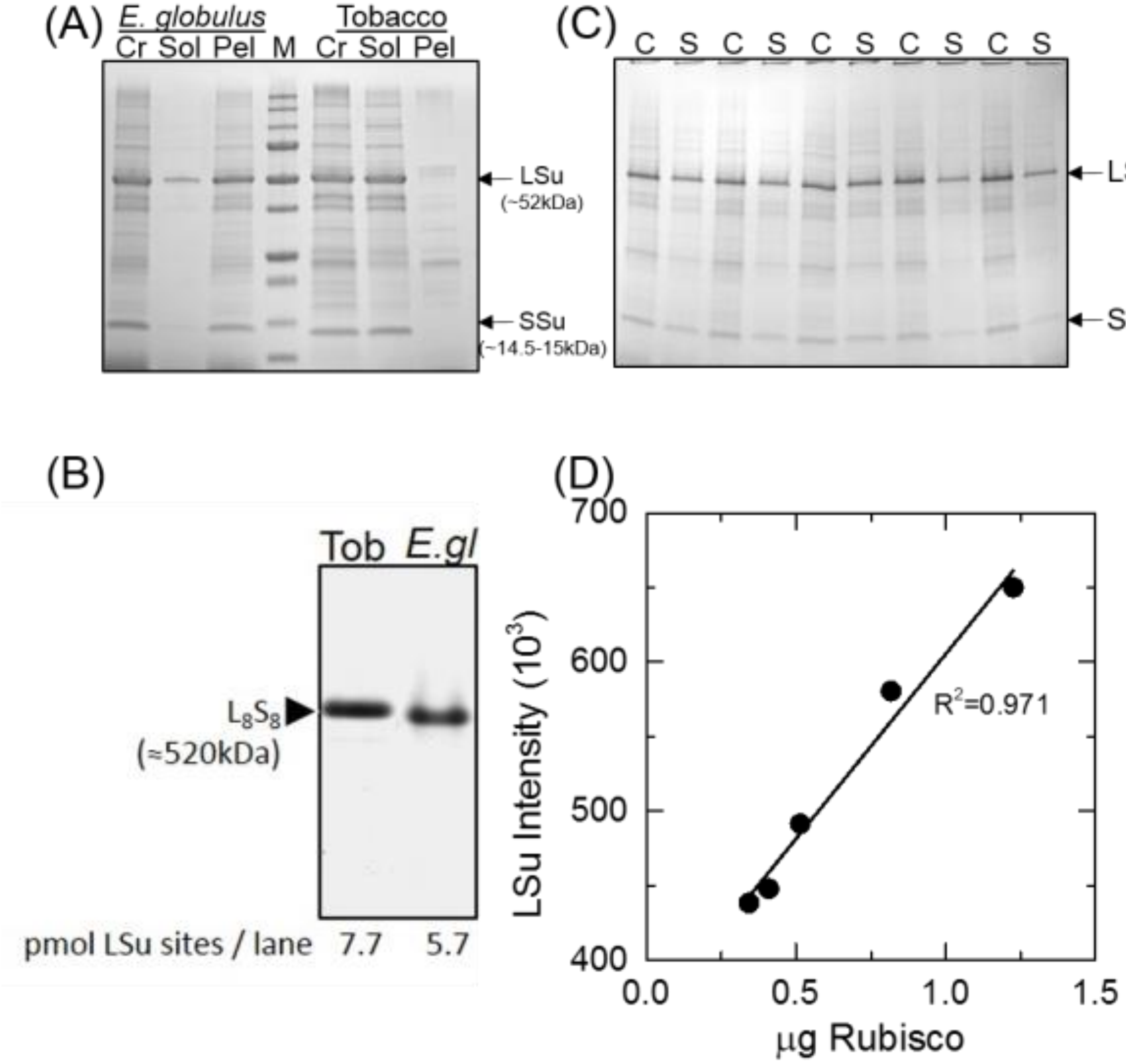

Supplementary Figure S2: Relative content (per leaf area) of the thylakoid complex PSII grown at ambient (aC) or elevated (eC) atmospheric [CO_2], and at ambient (aT) or elevated (eT) air temperature. PsbA contents were normalised relative to those in the aCeT samples *within each canopy*. Leaf PSII content were quantified relative to serial dilutions of a universal PsbA (D1) standard (see blot on the right). Values represent averages of 2-3 biological replicates ± SE (n=3 except for eCaT, where n=2).

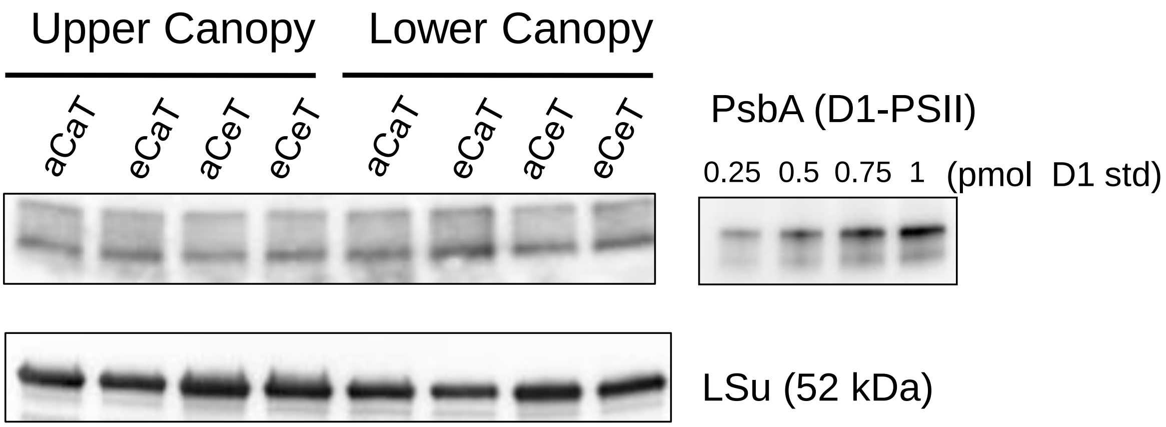

Supplement: supplementary_figures_S1_S2 [file erw484_suppl_supplementary_figures_S1_S2.pdf]
